# PTEN Modulates miR-21 Processing via RNA-Regulatory Protein RNH1

**DOI:** 10.1371/journal.pone.0028308

**Published:** 2011-12-05

**Authors:** Youn-Jae Kim, Se-Jeong Park, Eun Young Choi, Sol Kim, Hee Jin Kwak, Byong Chul Yoo, Heon Yoo, Seung-Hoon Lee, Daesoo Kim, Jong Bae Park, Jong Heon Kim

**Affiliations:** 1 Department of Biological Sciences, Korea Advanced Institute of Science and Technology, Daejeon, Korea; 2 Carcinogenesis Branch, Research Institute, National Cancer Center, Ilsan, Korea; 3 Specific Organs Cancer Branch, Research Institute, National Cancer Center, Ilsan, Korea; 4 Colorectal Cancer Branch, Research Institute, National Cancer Center, Ilsan, Korea; Instituto Nacional de Câncer, Brazil

## Abstract

Aberrant miR-21 expression is closely associated with cell proliferation, anti-apoptosis, migration, invasion, and metastasis in various cancers. However, the regulatory mechanism of miR-21 biogenesis is largely unknown. Here, we demonstrated that the tumor suppressor PTEN negatively regulates the expression of oncogenic miR-21 at the post-transcriptional level. Moreover, our results suggest that PTEN plays such a role through the indirect interaction with the Drosha complex. To elucidate how PTEN regulates pri- to pre-miR-21 processing, we attempted to find PTEN-interacting proteins and identified an RNA-regulatory protein, RNH1. Using the sensor to monitor pri-miR-21 processing, we demonstrated that RNH1 is necessary and sufficient for pri-miR-21 processing. Moreover, our results propose that the nuclear localization of RNH1 is important for this function. Further analysis showed that RNH1 directly interacts with the Drosha complex and that PTEN blocks this interaction. Taken together, these results suggest that the PTEN-mediated miR-21 regulation is achieved by inhibiting the interaction between the Drosha complex and RNH1, revealing previously unidentified role of PTEN in the oncogenic miR-21 biogenesis.

## Introduction

MicroRNAs (miRNAs) are small non-coding single-stranded RNAs approximately 22 nucleotides (nt) in length. They regulate the expression of most genes by partially binding to the 3′ untranslated regions of target mRNAs in a sequence-specific manner, where they act post-transcriptionally by destabilizing the target mRNAs, or inhibiting their translation, or both [1], [2]. Due to the gene regulatory function, miRNAs have been implicated in virtually all of cellular physiology, including development, cell growth, apoptosis, and differentiation. Moreover, many miRNAs have been reported as deregulated in diverse diseases including cancer and thereby have emerged as potential therapeutic targets [3], [4], [5], [6].

The biogenesis of miRNAs consists of several steps [7]. First, RNA polymerase II transcribes long pri-miRNAs. Next, the Drosha complex cleaves the pri-miRNAs into shorter pre-miRNAs in the nucleus. Then, the pre-miRNAs are transported to the cytoplasm by Exportin5, and the Dicer complex processes the pre-miRNAs into mature miRNAs. Recently, several studies have reported that miRNA biogenesis can be regulated post-transcriptionally as well as transcriptionally [8], [9], [10]. For instance, BMP4 or TGF-β promoted the Drosha-mediated cleavage of pri-miRNAs by stimulating the binding of SMAD to the Drosha complex [11], whereas estrogen receptor α inhibited the pri-miRNA processing through the interaction with the Drosha complex under estrogen treatment [12]. In addition, KH-type splicing regulatory protein (KSRP) enhanced both Drosha and Dicer processing [13]. p53, a tumor suppressor, also stimulated the processing of pri-miRNAs via binding to the Drosha complex under conditions of DNA damage [14].

MicroRNA-21 (miR-21) has been shown to be overexpressed in almost all types of cancer [15], [16]. The overexpression of miR-21 in these cancers is associated with cell proliferation, anti-apoptosis, migration, invasion, and metastasis. These studies imply that miR-21 plays key oncogenic roles in cancer initiation and progression, and thereby it has been classed as an oncomir. Thus, it is very important to understand how the expression of miR-21 is deregulated in cancers. The deregulation of miR-21 expression has not been associated with its gene amplification in most cancers [16], implying that the overexpression of miR-21 is caused transcriptionally or post-transcriptionally or both. Recently, we have observed that PTEN, a tumor suppressor frequently mutated in many cancers, inhibits miR-21 expression in glioblastoma cell lines [17], suggesting that the aberrant expression of miR-21 may be associated with the functional status of PTEN. However, its detailed inhibitory mechanisms remain elusive. Here we show how PTEN regulates the biogenesis of miR-21. Our results indicate that PTEN modulates miR-21 synthesis at the post-transcriptional level.

## Results

### PTEN regulates miR-21 biogenesis at the post-transcriptional levels

**Figure 1:**
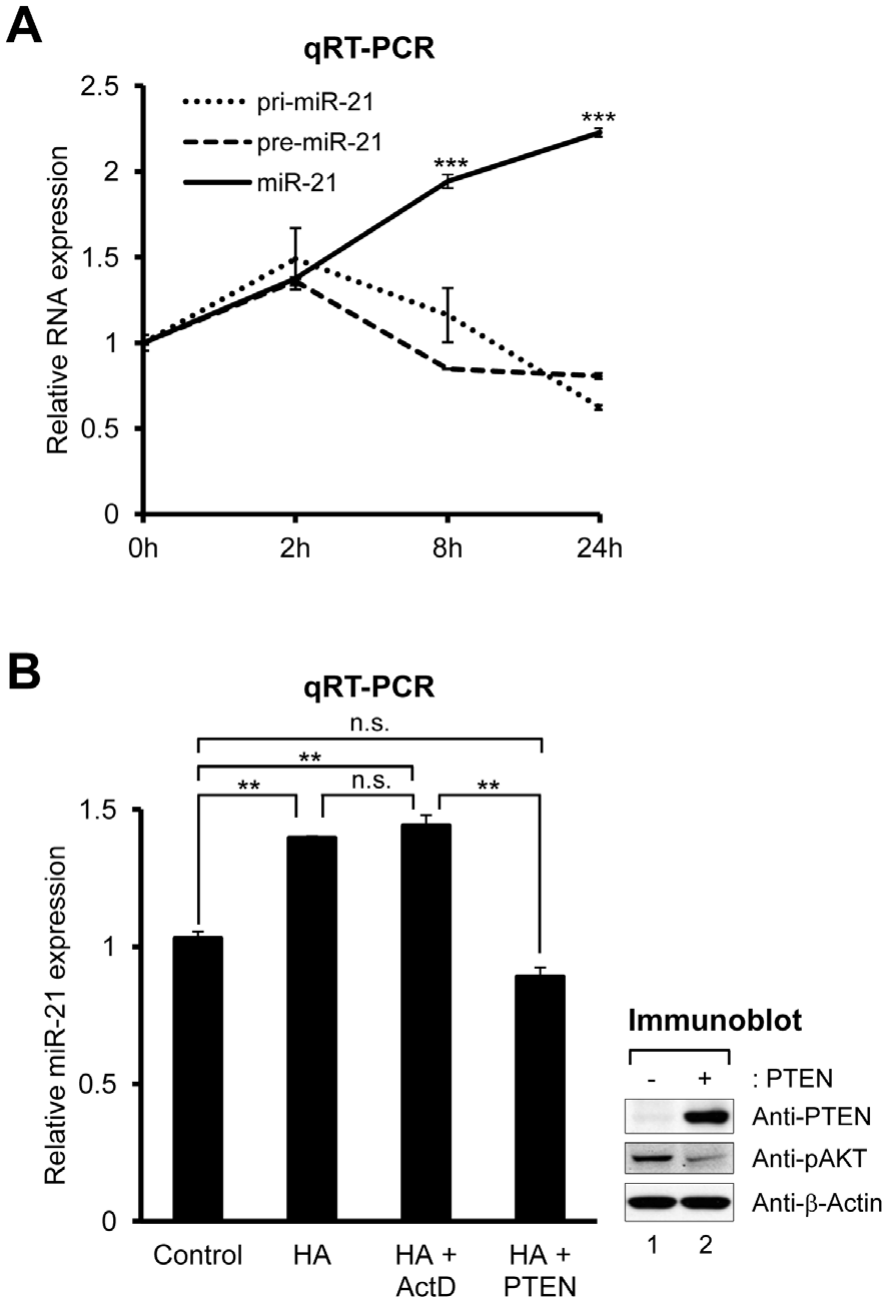
Post-transcriptional regulation of miR-21 expression by PTEN. (A) Time course of pri- (small dotted line), pre- (large dotted line), or mature miR-21 (single unbroken line) expression in U87MG cells after HA treatment. Expression level of each miRNA was analyzed by qRT-PCR reactions which were normalized to GAPDH for pri- and pre-miR-21, and U6 small nuclear RNA for mature miR-21. (B) Expression level of mature miR-21. U87MG cells pretreated with ActD or overexpressing PTEN were treated with HA for 24 hr. Data represent the mean values of at least three independent experiments performed in triplicate. **P<0.01 and ***P<0.001; n.s., not significant; Student's t test. Error bars indicate s.e.m. PTEN expression and AKT phosphorylation were confirmed by immunoblotting.

Previously, we have shown that hyaluronan (HA) increases miR-21 expression, which facilitates glioblastoma invasion [17]. To examine whether the regulation of HA-induced miR-21 biogenesis is transcriptional or post-transcriptional, we measured pri-, pre-, and mature miR-21 at different times after HA treatment in U87MG cells (Fig. 1A). Pri- and pre-miR-21 were hardly altered, whereas mature miR-21 was significantly increased, implying that the miR-21 is regulated in the post-transcriptional processing steps of miRNA biogenesis. To verify that HA does not affect the transcription of miR-21, we used a transcription inhibitor, actinomycin D (ActD). ActD did not affect mature miR-21 expression by HA (Fig. 1B), supporting the conclusion that HA-induced miR-21 expression occurs at the post-transcriptional levels. Moreover, the up-regulation of miR-21 by HA was suppressed by the overexpression of PTEN (Fig. 1B), as we previously reported [17]. Thus, these results suggest that PTEN regulates miR-21 biogenesis at the post-transcriptional levels.

### PTEN regulates pri-miR-21 processing

**Figure 2:**
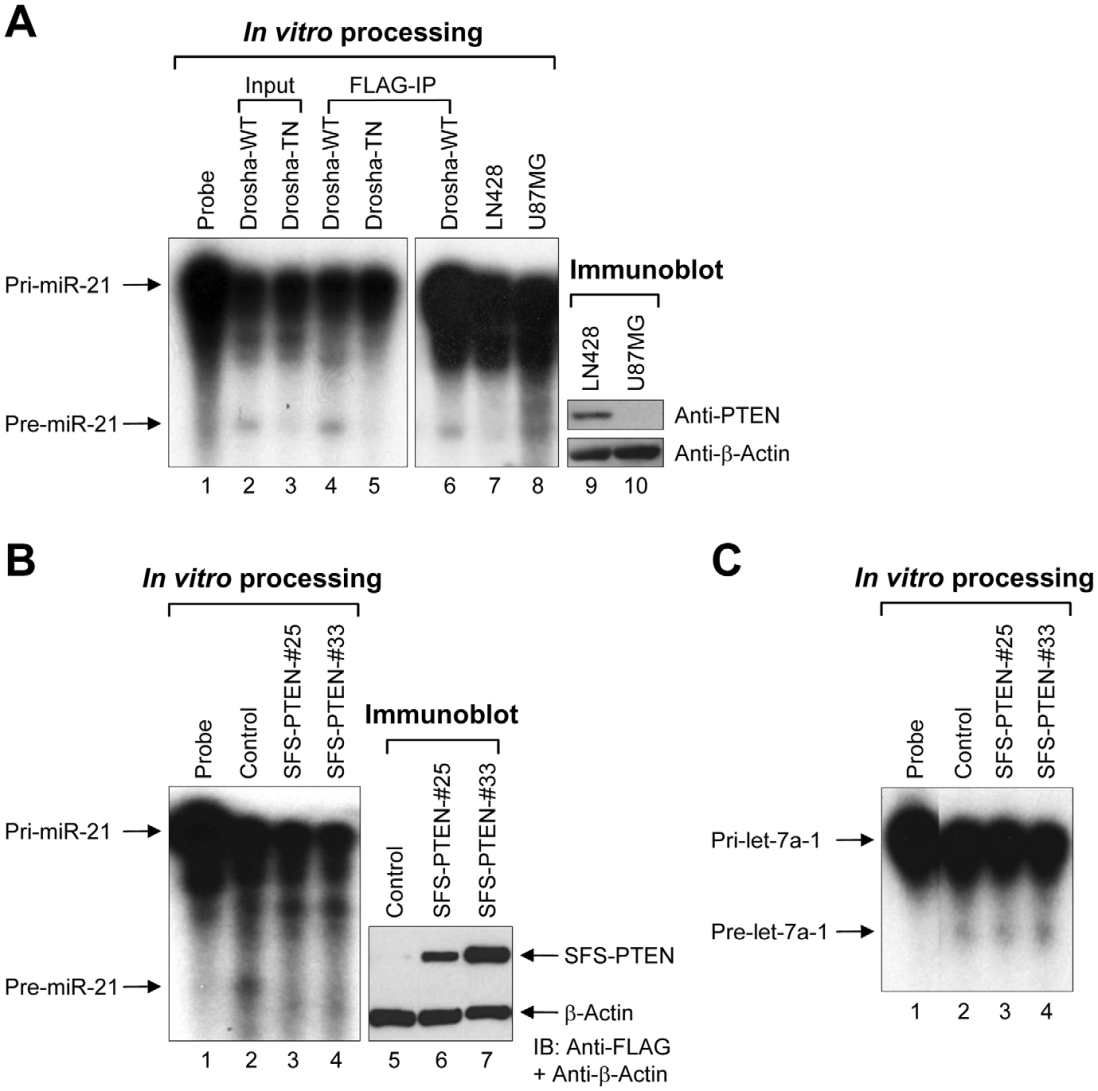
PTEN regulates pri-miR-21 processing. (A) *In vitro* pri-miR-21 processing with Drosha-expressing cell lysates, Drosha immunoprecipitates, or glioblastoma cell lysates. Lane 1: probe only; lane 2: Drosha-WT overexpressed cell lysates; lane 3: Drosha-TN overexpressed cell lysates; lane 4: Drosha-WT immunoprecipitate; lane 5: Drosha-TN immunoprecipitate; lane 6: Drosha-WT immunoprecipitate; lane 7: LN428 cell lysates; lane 8: U87MG cell lysates. PTEN expression of LN428 (lane 9) and U87MG (lane 10) was confirmed by immunoblotting. (B) *In vitro* pri-miR-21 processing with parental 293T and PTEN expressing cell lysates. Lane 1: probe only; lane 2: parental 293T cell lysates; lane 3: PTEN expressing cell (clone #25) lysates; lane 4: PTEN expressing cell (clone #33) lysates. PTEN expression of parental 293T (lane 5) and PTEN expressing cells (lane 6: clone #25; lane 7: clone #33) was confirmed by immunoblotting. (C) *In vitro* pri-let-7a-1 processing with parental 293T and WT-PTEN expressing cell lysates. Lane 1: probe only; lane 2: parental 293T cell lysates; lane 3: PTEN expressing cell (clone #25) lysates; lane 4: PTEN expressing cell (clone #33) lysates. IB; immunoblot.

To directly test whether PTEN affects the biogenesis of miR-21 at the post-transcriptional level, we performed an *in vitro* pri-miRNA processing assay. Drosha was FLAG-tagged on its carboxyl terminus and was ectopically expressed in 293T cells. As shown in Fig. 2A, the Drosha complex converted [^32^P]-labeled pri-miR-21 to pre-miR-21. Using this assay system, we examined whether the presence of PTEN affects miR-21 processing. Whole cell extracts were prepared from LN428 cells harboring wild-type (WT) PTEN or U87MG cells lacking functional PTEN. When radiolabeled pri-miR-21 was added into the whole cell extracts, pre-miR-21 levels were lower in LN428 cell extracts than in U87MG ones (Fig. 2A), implying that the cellular status of PTEN may affect pri-miR-21 processing. To further demonstrate this, whole cell extracts were prepared from parental 293T cells and two PTEN-overexpressing 293T stable cell lines (#25 and #33), and the pri- to pre-miRNA processing activities were evaluated using these extracts. Similar results were obtained compared to parental 293T cell extracts (Fig. 2B). The parental 293T cell extracts processed pri- to pre-miR-21, but PTEN-overexpressed 293T cell extracts did not. However, PTEN overexpression had little effect on pri-let-7a-1 processing (Fig. 2C), indicating that PTEN may preferentially regulate pri-miR-21 processing.

Pri-miRNA is processed to precursor one by the Drosha complex. To regulate miRNA processing, PTEN may interact with this complex. To test this possibility, we next investigated the molecular interaction between the Drosha complex and PTEN through co-immunoprecipitation (co-IP) experiments. After the expression of FLAG-PTEN in 293T cells, the cell extracts were immunoprecipitated using FLAG antibody. However, we could not detect the endogenous Drosha in the FLAG-PTEN immunoprecipitate by Drosha specific antibody (data not shown), suggesting that PTEN indirectly regulates pri-miR-21 processing.

### PTEN interacts with the RNA-regulatory protein RNH1, which is potentially involved in miR-21 biogenesis

**Figure 3:**
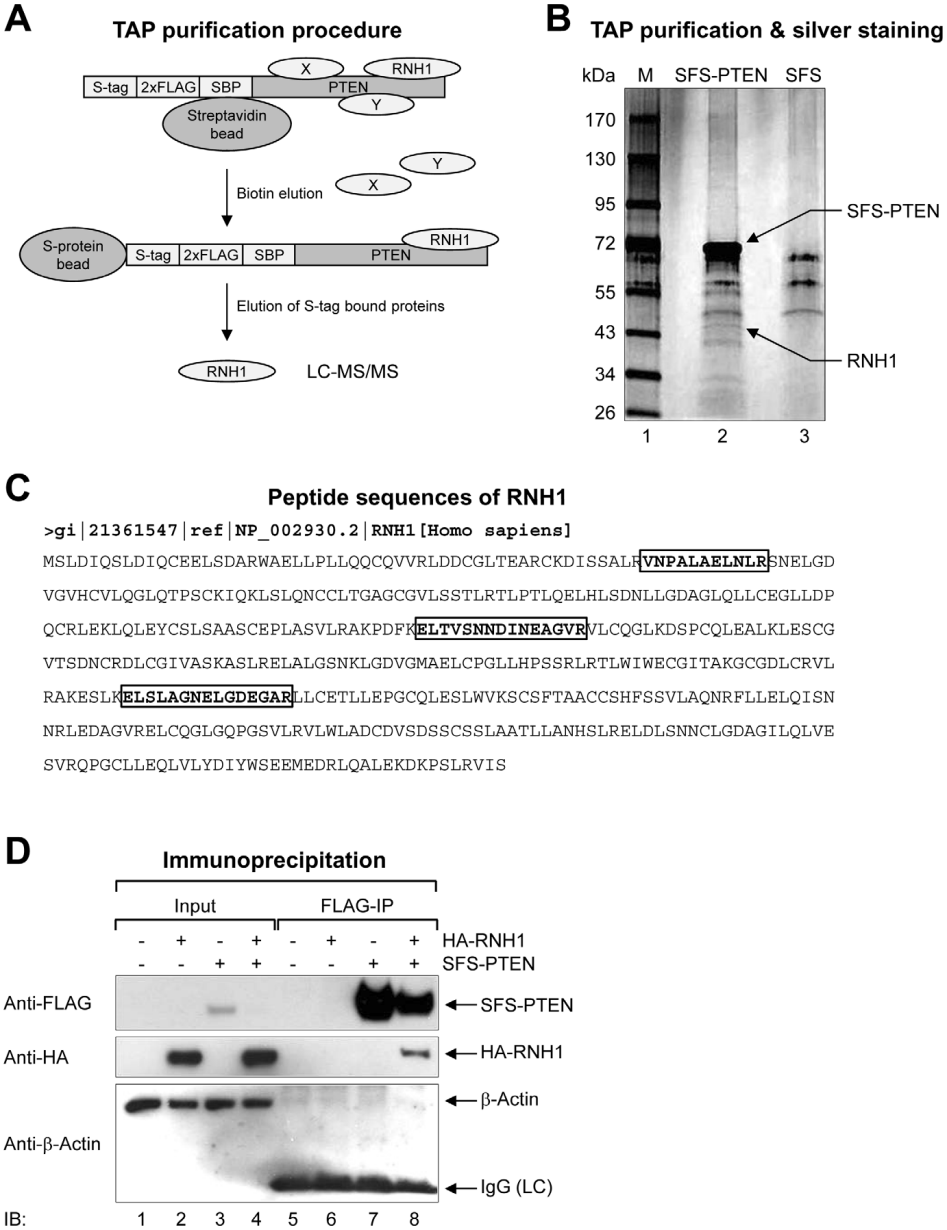
Identification of RNH1 as a novel PTEN-interacting protein. (A) Schematic diagram of tandem affinity purification (TAP) procedure. A S-tag, double FLAG tag, and streptavidin-binding peptide were fused at the N-terminus of PTEN (SFS-PTEN). After sequential streptavidin and S-protein bead binding, the PTEN-interacting proteins were eluted from S-protein beads. The eluted proteins were analyzed by LC-MS/MS. X and Y represent nonspecifically interacting proteins. (B) The silver staining result of TAP purified proteins. Arrows indicate an RNA-regulatory protein RNH1 (lower) and SFS-PTEN (upper). (C) Peptide sequences (boxes) of RNH1 by LC-MS/MS analysis. (D) Physical interaction between PTEN and RNH1. Plasmids encoding HA-RNH1 and SFS-PTEN were ectopically expressed in 293T cells. SFS-PTEN was precipitated with anti-FLAG M2 affinity agarose gels and then immunoblotting with anti-FLAG, anti-HA, or anti-β-Actin (negative binding control) was performed. IgG (LC): IgG (light chain).

If the regulation of pri-miR-21 processing by PTEN is indirect, it is possible that PTEN acts through another mediator. To identify the binding partners of PTEN that may be directly involved in pri-miRNA processing, we purified PTEN-containing complexes from 293T cells stably expressing PTEN with an N-terminal tandem affinity purification tag consisting of an S-tag, double FLAG epitopes, and a streptavidin-binding peptide (SFS-PTEN). An advantage of this method is that real quantitative determination of protein partners *in vivo* is possible without prior knowledge of the composition of the complex [18], [19]. After two successive affinity purifications, the chance for contaminants to be retained in the first eluent is significantly reduced. We detected several specific bands that eluted with the SFS-PTEN, but not with the SFS itself (Fig. 3B). However, mass spectrometry revealed that one band among those analyzed was a candidate PTEN-interacting partner, RNH1 (ribonuclease/angiogenin inhibitor 1) (Fig. 3B and 3C). The interaction between PTEN and RNH1 was confirmed using a co-IP experiment (Fig. 3D). 293T cells were transfected with plasmids encoding SFS-PTEN and hemagglutinin (HA)-tagged RNH1; FLAG IP and immunoblotting were then performed. As expected, HA-RNH1 was co-precipitated with SFS-PTEN (Fig. 3D).

### Construction of sensors to monitor pri-miR-21 processing

**Figure 4:**
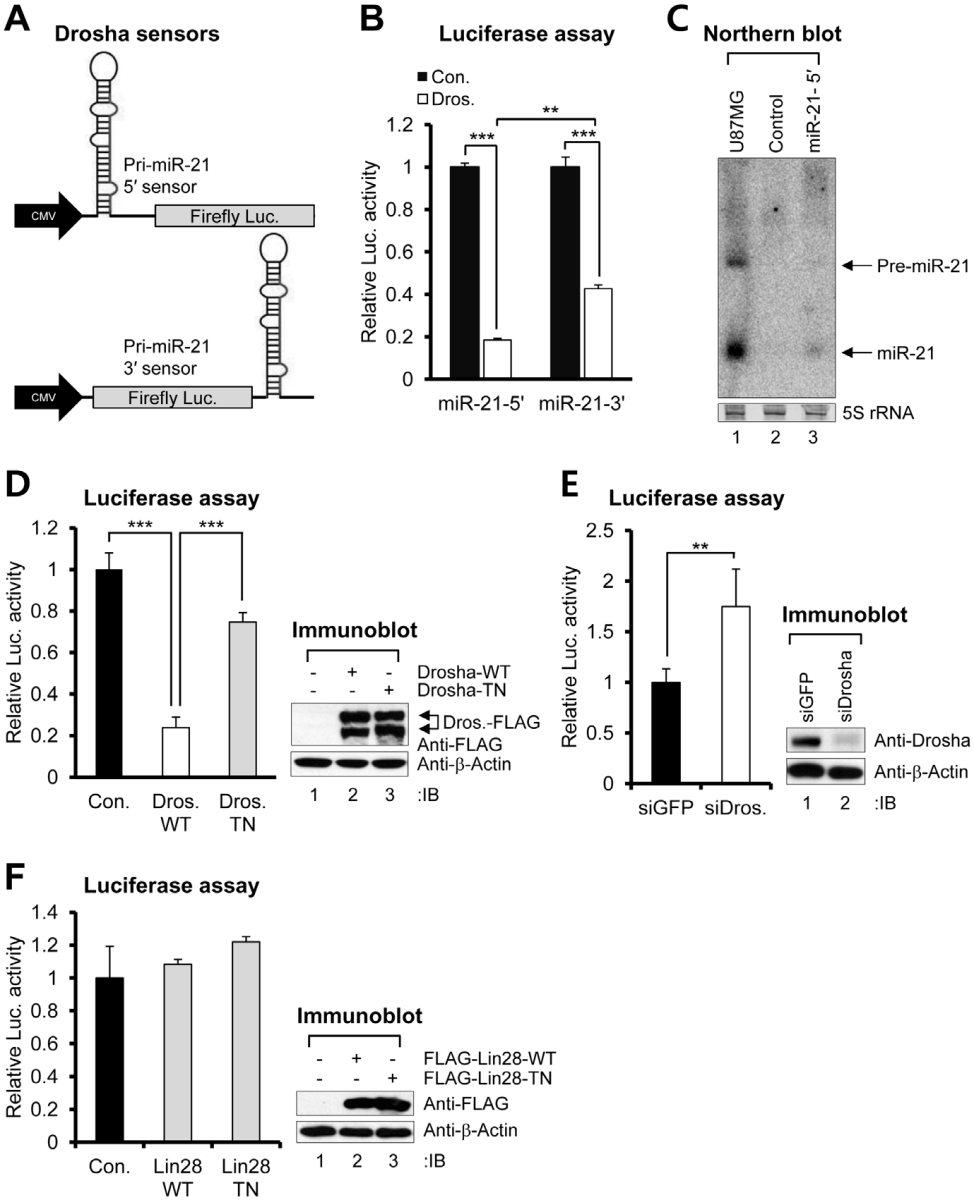
Establishment of pri-miR-21 processing sensors. (A) Schematic depiction of pri-miR-21 processing sensors. (B) Luciferase assay results of each transfectant. The sensors or control plasmid were ectopically expressed along with the plasmid encoding Drosha (Dros.)-WT in 293T cells. (C) Northern blot analysis of sensor. Total RNAs from U87MG cells were used as positive controls of pre- and mature miR-21 expression. The control plasmid or 5′-sensor was ectopically expressed in 293T cells. Arrows indicate positions of pre- (upper) and mature (lower) miR-21. 5S rRNA was used as the loading control. (D) Luciferase assay results of each transfectant. The sensor was co-transfected into 293T cells along with the plasmid encoding Drosha-WT or Drosha-TN. Drosha expression was confirmed by immunoblotting. (E) Luciferase assay results of each transfectant. The sensor was co-transfected into 293T cells along with siRNA against green fluorescent protein (GFP) or Drosha. Drosha expression was confirmed by immunoblotting. (F) Luciferase assay results of each transfectant. The sensor was co-transfected into 293T cells along with the plasmid encoding FLAG-Lin28-WT or FLAG-Lin28-TN which has no binding activity for the pre-let-7 family. Data represent the mean values of at least three independent experiments performed in triplicate. **P<0.01, and ***P<0.001; Student's t test. Error bars indicate s.e.m.

To test the possibility of pri-miR-21 processing regulation by RNH1, we designed luciferase assay systems to act as “sensors” to monitor pri-miRNA processing [20]. These sensors were constructed as such that 212 nt genomic segment of pri-miR-21 with pre-miR-21 sequences in its center was inserted into the 5′ or 3′ end of the firefly luciferase gene (Fig. 4A). If the Drosha complex cleaves the pri-miR-21 portion of these sensors, the luciferase transcripts would become unstable, resulting in decreased translation efficiency. Indeed, co-transfection of the sensors and the Drosha-WT construct into 293T cells exhibited lower luciferase activity compared to the control (Fig. 4B and 4D). In addition, the luciferase activity of the 5′-sensor was even more diminished by the overexpression of Drosha-WT than was the activity of the 3′-sensor (Fig. 4B), demonstrating that the 5′-sensor can monitor pri-miR-21 processing more sensitively than the 3′-sensor. Thus, we used the 5′-sensor in all of the following experiments and simply describe it as the “sensor”. Northern blotting revealed that the sensor is normally processed to pre- and mature miR-21 (Fig. 4C). To test the specificity of the construct, the sensor was co-transfected with a catalytically dead, trans-dominant negative (TN) Drosha mutant. The Drosha-TN did not exhibit significantly diminished luciferase activity compared to Drosha-WT (Fig. 4D). Moreover, the knockdown of Drosha by specific siRNA increased the activity of the sensor (Fig. 4E), suggesting that the sensor can specifically recapitulate pri-miR-21 processing and can identify both positive and negative regulators of pri-miR-21 processing by the Drosha complex.

### The novel PTEN-binding protein RNH1 modulates pri-miR-21 processing

**Figure 5:**
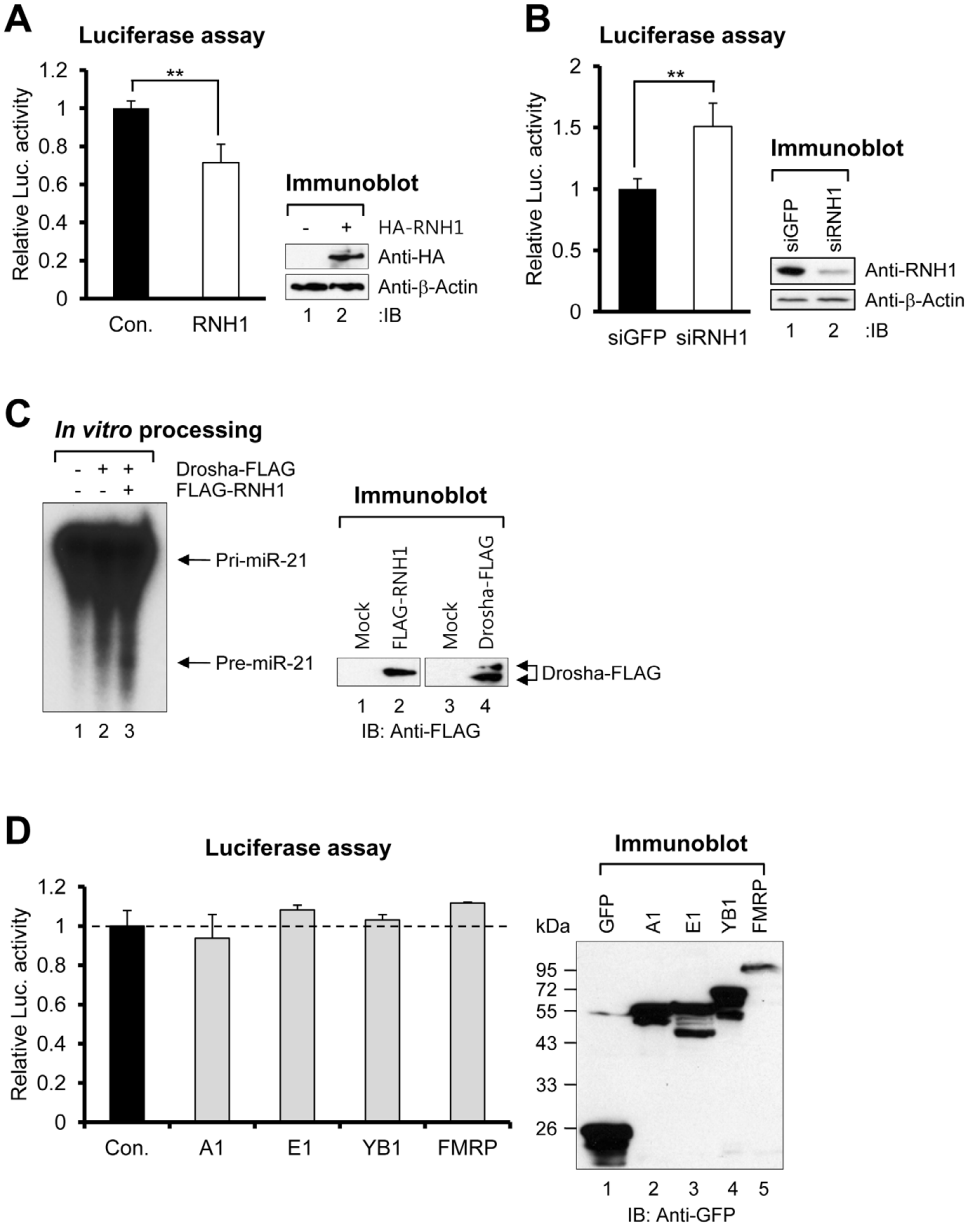
RNH1 modulates pri-miR-21 processing. (A) Luciferase assay results of each transfectant. The sensor was co-transfected into 293T cells along with the plasmid encoding HA-RNH1. HA-RNH1 expression was confirmed by immunoblotting. (B) Luciferase assay results of each transfectant. The sensor was co-transfected into 293T cells along with siRNA against GFP or RNH1. (C) *In vitro* pri-miR-21 processing with Drosha or RNH1 immunoprecipitates. The expression of FLAG-RNH1 and Drosha-FLAG was confirmed by immunoblotting. (D) Luciferase assay results of each transfectant. The sensor was co-transfected into 293T cells along with the plasmid encoding GFP, GFP-hnRNP A1 (A1), GFP-hnRNP E1 (E1), GFP-YB1 (YB1), or GFP-FMRP (FMRP). Each expression was confirmed by immunoblotting. Data represent the mean values of at least three independent experiments performed in triplicate. **P<0.01; Student's t test. Error bars indicate s.e.m.

Using the sensor, we examined whether the novel PTEN-binding protein RNH1 modulates pri-miR-21 processing. Overexpressed RNH1 promoted the processing of pri-miR-21 (Fig. 5A), and knockdown of RNH1 by siRNA suppressed the processing of pri-miR-21 (Fig. 5B). We hypothesized that RNH1 could directly facilitate pri-miR-21 processing. To examine the possibility, we tested whether RNH1 promotes pri-miR-21 processing *in vitro*. To facilitate the processing reaction, purified Drosha-FLAG was co-incubated with purified FLAG-RNH1 and radiolabeled pri-miR-21. We observed that the FLAG immunoprecipitate containing FLAG-RNH1 potentially activated the processing of pri-miR-21 *in vitro* (Fig. 5C). These results suggest that RNH1 is necessary and sufficient for pri-miR-21 processing. In contrast, other RNA regulatory proteins, such as hnRNP A1, hnRNP E1, YB1, and FMRP, had little effect on the processing (Fig. 5D). Among these proteins, hnRNP A1 is known to be specifically required for pri-miR-18a and pri-let-7a-1 processing [21], [22]. Moreover, Lin28, which specifically regulates the biogenesis of let-7 [23], [24], [25], [26], did not affect the processing of pri-miR-21 (Fig. 4F). Taken together, these results suggest that RNH1 specifically regulates pri-miR-21 processing.

### Nuclear RNH1 facilitates pri-miR-21 processing

**Figure 6:**
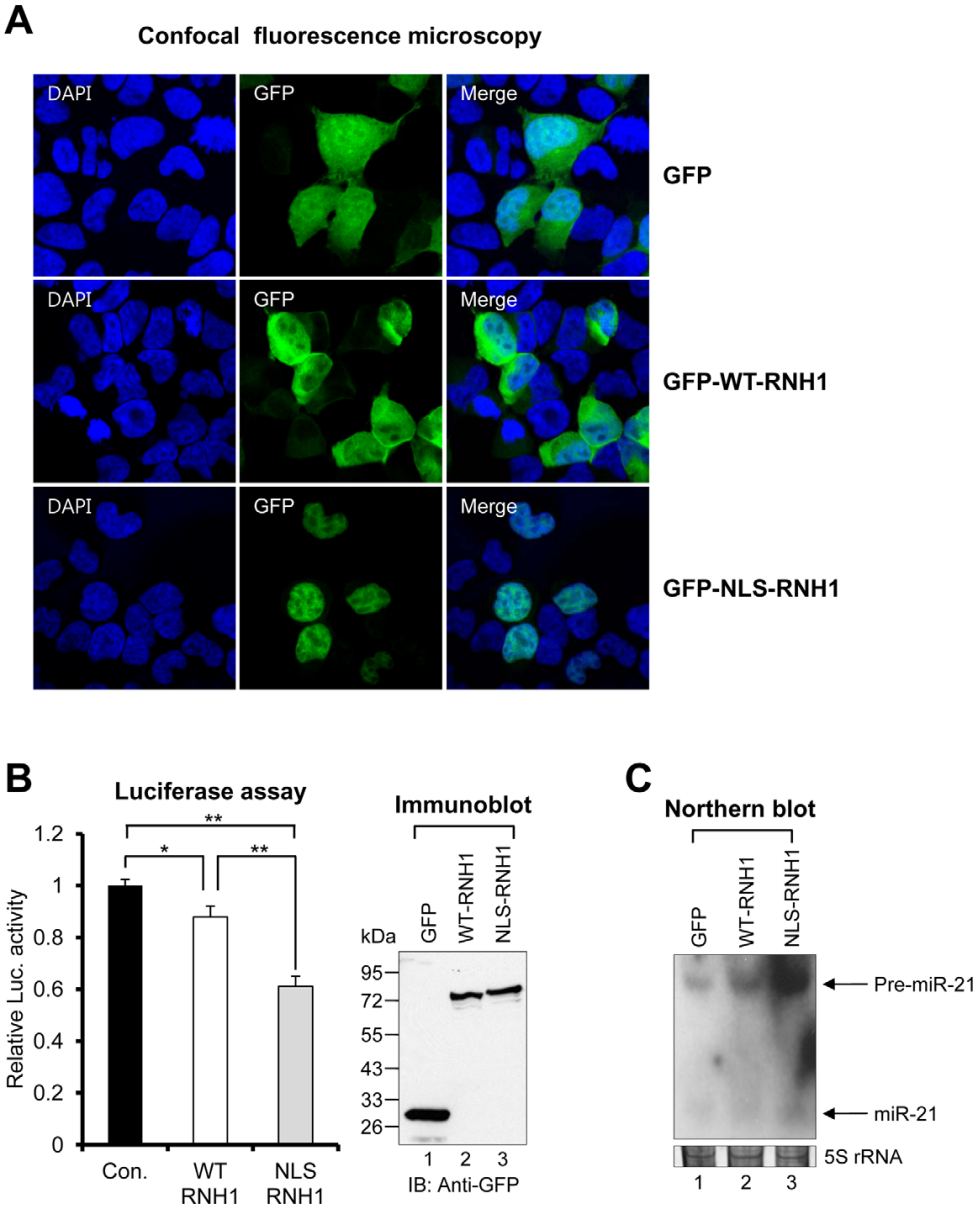
Nuclear localization of RNH1 is essential for the pri-miR-21 processing. (A) Confocal fluorescence microscopy of each transfectant. The plasmid encoding GFP, GFP-WT-RNH1, or GFP-NLS-RNH1 was ectopically expressed in 293 cells. The experiments were repeated at least three times with similar results. (B) Luciferase assay results of each transfectant. The sensor was co-transfected into 293T cells along with the plasmid encoding GFP, GFP-WT-RNH1 (WT-RNH1), or GFP-NLS-RNH1 (NLS-RNH1). Data represent the mean values of at least three independent experiments performed in triplicate. *P<0.05, **P<0.01; Student's t test. Error bars indicate s.e.m. Each protein expression was confirmed by immunoblotting. (C) Northern blot analysis of each transfectant. The plasmid encoding GFP, WT-RNH1, or NLS-RNH1 was ectopically expressed in 293T cells along with the plasmid encoding the pri-miR-21 minigene. Arrows indicate positions of pre- (upper) and mature (lower) miR-21. 5S rRNA was used as the loading control. The experiments were repeated at least three times with similar results.

As mentioned earlier, miRNA processing by the Drosha complex occurs inside the nucleus. Thus, if RNH1 directly regulates the function of the Drosha complex, it should reside in the nucleus. In fact, a previous report has shown that RNH1 is present not only in the cytoplasm, but also in the nucleus [27]. In agreement with the report, GFP-WT-RNH1 was localized in the nucleus as well as in the cytoplasm (Fig. 6A). To examine the role of nuclear RNH1 in pri-miR-21 processing, we generated an RNH1 molecule that contained a nuclear localization signal (NLS) at the amino terminus (NLS-RNH1). GFP-NLS-RNH1 was localized exclusively in the nucleus. If the role of RNH1 mainly is involved in pri- to pre-miR-21 processing, NLS-RNH1 would be expected to enhance the processing activity of the Drosha complex. As predicted, cells expressing NLS-RNH1 display higher levels of pri-miR-21 processing than cells expressing WT-RNH1 (Fig. 6B). Consistent with these results, Northern blot analysis confirmed that pri-miR-21 processing was more increased in the presence of NLS-RNH1 (Fig. 6C).

### PTEN inhibits the interaction of RNH1 with the Drosha complex

**Figure 7:**
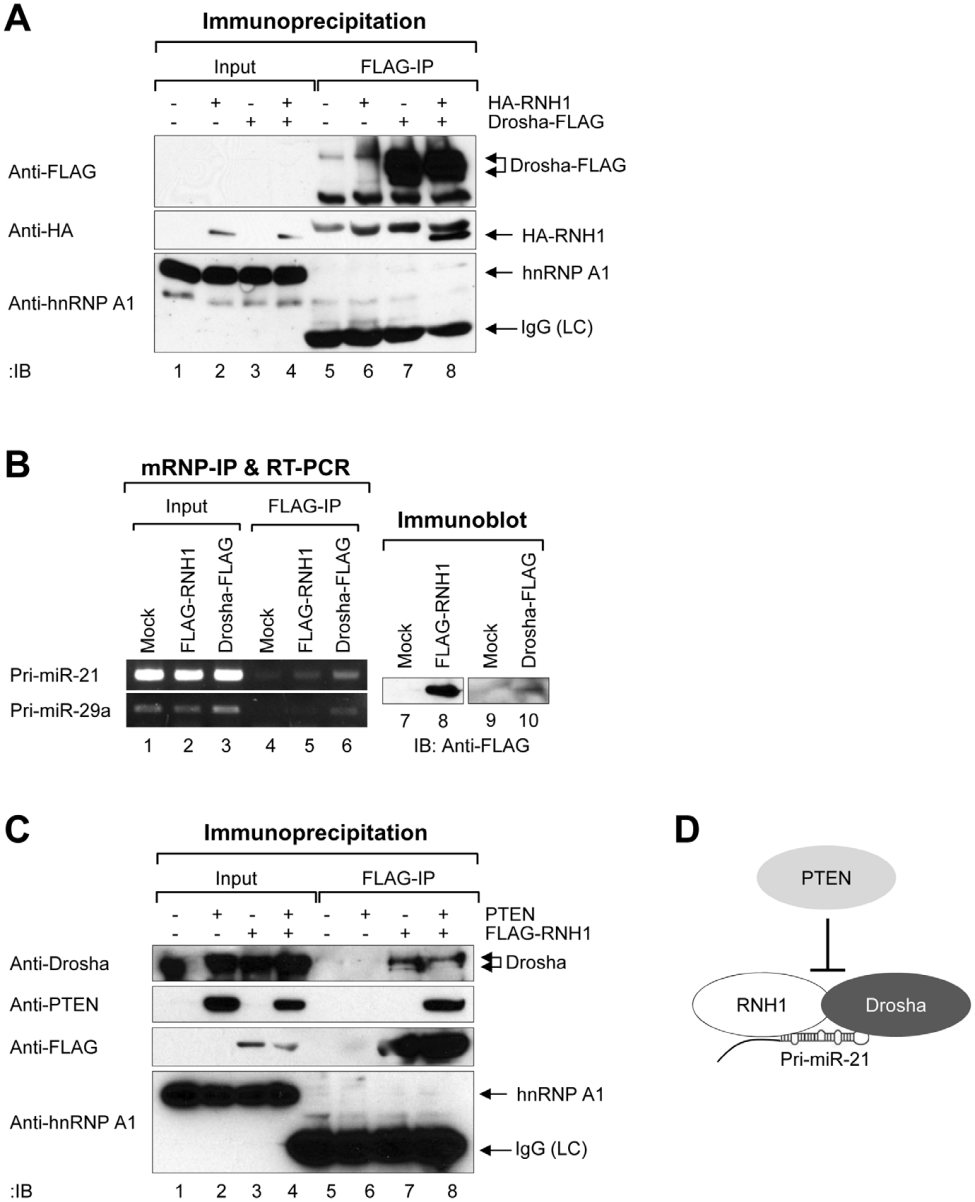
PTEN has inhibitory role for RNH1 interaction to the Drosha complex. (A) Physical interaction between RNH1 and the Drosha complex. The plasmid encoding Drosha-FLAG and HA-RNH1 were ectopically expressed in 293T cells. Drosha-FLAG was precipitated with anti-FLAG M2 affinity agarose gels and then and then immunoblotting with anti-FLAG (antibody 4C5), anti-HA, or anti-hnRNP A1 (negative binding control) was performed. (B) Physical interaction between RNH1 and pri-miR-21. The plasmid encoding FLAG-RNH1 or Drosha-FLAG was ectopically expressed in HeLa cells. RNA-protein complexes were precipitated with anti-FLAG M2 affinity agarose gels. After IP of RNA-protein complexes, RNAs were isolated and used in RT-PCR reactions with specific oligomers for pri-miR-21 or pri-miR-29a. The PCR products were resolved on 1% agarose gel. Each protein expression was confirmed by immunoblotting. (C) The inhibitory role of PTEN in the interaction of RNH1 with the Drosha complex. The plasmid encoding FLAG-RNH1 and PTEN were ectopically expressed in 293T cells. FLAG-RNH1 was precipitated with anti-FLAG M2 affinity agarose gels and then immunoblotting with anti-Drosha, anti-PTEN, anti-FLAG (anti-DYKDDDDK), or anti-hnRNP A1 (negative binding control) was performed. The experiments were repeated at least three times with similar results. (D) Proposed illustration shows that PTEN negatively modulates the biogenesis of miR-21 by blocking the interaction of the Drosha complex with RNH1, which facilitates pri-miR-21 processing.

If RNH1 functions as the regulator of miRNA processing in the nucleus, it may interact with the Drosha complex. To test this hypothesis, we performed co-IP experiments for RNH1 and Drosha. After the co-expression of HA-RNH1 and Drosha-FLAG in 293T cells, the Drosha-FLAG in the cell extracts was immunoprecipitated using FLAG antibody. The results demonstrated that RNH1 binds to the Drosha complex (Fig. 7A), implying that RNH1 regulates pri-miR-21 processing through the direct interaction with the Drosha complex.

Recently, RNA-regulatory proteins, such as hnRNP A1 and KSRP, were shown to bind directly to pri-miRNAs for facilitating the Drosha processing [13], [21], [22]. It is also plausible that RNH1 can regulate pri-miR-21 through the direct interaction. To test whether RNH1 can directly bind to pri-miR-21, we performed mRNP-IPs with FLAG antibody against FLAG-RNH1 or control Drosha-FLAG. Then the bound pri-miRNAs were analyzed by semi-quantitative RT-PCR with specific primers that amplify pri-miR-21 or the control pri-miR-29a. We could amplify pri-miR-21, but not pri-miR-29a, except in the Drosha-FLAG IP (Fig. 7B), indicating that RNH1 binds specifically to pri-miR-21 before the Drosha cleavage.

Above we showed that PTEN suppresses pri-miR-21 processing and suggested that RNH1 may be its mediator. Thus, we examined whether PTEN affects the interaction between RNH1 and Drosha. When PTEN was overexpressed, the interaction between RNH1 and Drosha was less apparent (Fig. 7C). Taken together, these results propose a model in which PTEN negatively modulates pri-miR-21 processing by blocking the RNH1-mediated recruitment of pri-miR-21 to the Drosha complex (Fig. 7D).

## Discussion

In this study, we showed that the well-known tumor suppressor PTEN inhibits the biogenesis of oncogenic miR-21 post-transcriptionally, revealing a previously unidentified function of PTEN. PTEN is frequently mutated or expressed at a diminished level in a wide variety of cancers [28], [29]. Our results imply that the malfunction of PTEN may be one cause for the increased expression of miR-21 in many cancers. Conversely, it has been reported that miR-21 negatively regulates PTEN by binding to its 3′UTR [30], [31]. Combined with our results, this suggests the possibility of a double-negative feedback loop between PTEN and miR-21 like that of Lin28 and let-7 [25], [32].

PTEN is mainly known as a lipid phosphatase, dephosphorylating phosphatidylinositol-3,4,5-trisphosphate 3-phosphatase (PIP_3_) into phosphatidylinositol-4,5-bisphosphate (PIP_2_) and thereby antagonizing the oncogenic PI3K/AKT pathway. Previously, we have reported that HA-induced miR-21 expression is AKT-independent [17]. Thus, the miR-21 regulation by PTEN may occur independently of its lipid phosphatase activity. Indeed, recent studies have reported that PTEN mediates other functions through protein-protein interactions [33], [34], [35], [36], [37]. Here, we identified a novel PTEN-interacting protein, RNH1, by tandem affinity purification. Our results suggest that PTEN regulates pri-miR-21 processing through the protein-protein interaction with RNH1.

Since the Drosha processing takes place in the nucleus, it is possible that PTEN modulates the miRNA-regulatory role of RNH1 in the nucleus. In fact, PTEN is mainly known to function as a lipid phosphatase on the inner surface of the plasma membrane, but nuclear functions of PTEN have also been reported [33], [34], [35], [36], [37]. Moreover, in normal cells, PTEN is mainly found in the nucleus rather than in the cytoplasm, suggesting that nuclear PTEN plays a tumor-suppressive role [38]. Indeed, it has been reported that nuclear PTEN is involved in chromosome stability, DNA repair, and cell cycle control [33], [36], [37], [38]. Although PTEN does not have a definite nuclear localization signal (NLS), several studies have reported that PTEN can be localized in the nucleus by various mechanisms [34], [35], [38]. Thus, it will be interesting to further evaluate whether nuclear PTEN plays a role in miRNA biogenesis. Alternatively, cytoplasmic PTEN may retain RNH1 in the cytoplasm through the protein-protein interaction, resulting in decreased levels of nuclear RNH1 and thereby reduced pri-miR-21 processing.

RNH1 contains fifteen leucine-rich repeats (LRRs), which consist of approximately 22–28 amino acids and are present in numerous proteins with diverse functions [39]. These LRRs are usually involved in the formation of protein-protein interactions. Thus, RNH1 may interact with PTEN or the Drosha complex through these LRRs. Moreover, our results suggest that RNH1 binds to pri-miR-21 as well. Indeed, the mRNA export factor TAP/hNXF1 binds to retroviral genomic RNA through the LRRs [40]. Therefore, RNH1 may bind directly to pri-miR-21 through the LRRs. However, it remains to be determined whether RNH1 regulates the processing of other miRNAs and whether the regulation is sequence specific.

We have shown that RNH1 physically associates with the Drosha complex. RNH1 is mainly known to function in the cytoplasm and inhibit RNases such as RNASE1, RNASE2, and angiogenin by forming high-affinity heterodimers with them [41]. Although the nuclear localization of RNH1 has also been reported [27], it is unknown if the nuclear RNH1 plays a role in miRNA biogenesis. Our results demonstrated that the enforced nuclear expression of RNH1 increases its activity promoting miRNA processing. In contrast to its reported RNase inhibitory role in the cytoplasm, these results suggest that RNH1 has a unique nuclear role involving activation of the RNase III Drosha for efficient miRNA processing.

Among a number of RNA-regulatory proteins, DEAD box RNA helicases p68 and p72 [42], hnRNP A1 [21], Lin28 [23], KSRP [13], Ars2 [43], [44], SF2/ASF [45] and MBNL1 [46] have already been reported as being able to modulate miRNA biogenesis at the post-transcriptional level. Moreover, recent studies on the interplay among them show the complexity of miRNA synthesis regulation [22], [46]. In addition to our present study, miR-21 processing has also been shown to be regulated by other proteins, such as SMAD [11], KSRP [13], and Ars2 [43], [44], adding another layer of complexity. Interestingly, KSRP [13] and Ars2 [43] facilitate the processing of tumor-suppressive let-7 as well as oncogenic miR-21, whereas we demonstrated that PTEN seems not to affect let-7 processing, implying that PTEN-dependent miRNA regulation may be specific to oncomirs.

In summary, we propose that PTEN suppresses pri- to pre-miR-21 processing through inhibiting the interaction of the Drosha complex with RNH1, an RNA-regulatory protein, which facilitates the processing. These results reveal another novel tumor-suppressive role of PTEN through blocking the biogenesis of oncogenic miR-21.

## Materials and Methods

### Reagents and cell culture

HA was purchased from Sigma-Aldrich and reconstituted in DMEM (HyClone). 293, 293T, HeLa, and glioblastoma cells (U87MG and LN428) were obtained from the American Type Culture Collection. The cells were cultured in DMEM supplemented with 10% fetal bovine serum (HyClone) and penicillin/streptomycin (HyClone).

### Construction of plasmids

For the generation of pSFS-PTEN, plasmid containing the full-length coding sequence of PTEN (pcDNA3-PTEN; gift of Y. E. Whang) was PCR amplified with the following oligomers specific for PTEN: sense, 5′-GGAATTCCATATGACAGCCATCATCAAAGAGATCG-3′ and antisense, 5′-CGG GATCCTCAGACTTTTGTAATTTGTGTATGCTG-3′. The amplified DNA was digested with *EcoR*I-*BamH*I and then inserted into pSFS [47]. For the generation of pCMV-HA-RNH1, plasmid containing the full-length cDNA of RNH1 (hMU010089; 21C Frontier Human Gene Bank, Korea), was PCR-amplified with the following oligomers specific for RNH1: sense, 5′-GGAATTCGGATGAT GAGCCTGGACATCCAG-3′ and antisense, 5′-CCGCTCGAGTCAGGAGATGACCCTCAG-3′. The amplified DNA was digested with *EcoR*I-*Xho*I and then inserted into pCMV-HA (Clontech). For the construction of pSG5L-RNH1 and pSG5L-NLS-RNH1, plasmid pCMV-RNH1 was PCR amplified with the following oligomers specific for RNH1: sense, 5′-CGGGATCCATGAGCCTGGACATCCA G-3′ and antisense, 5′-GGAATTCTCAGGAGATGACCCTCAG-3′. The amplified DNA was digested with *BamH*I-*EcoR*I and then inserted into pSG5L-PTEN and pSG5L-NLS-PTEN (Addgene) digested with *BamH*I-*EcoR*I, respectively. For the generation of pEGFPc1-WT-RNH1, pSG5L-RNH1 was digested with *BamH*I-*EcoR*I and then inserted into *Bgl*II-*EcoR*I digested pEGFPc1 (Clontech). pEGFPc1-NLS-RNH1 was generated by amplification of pSG5L-NLS-RNH1 by PCR with the following oligomers: sense, 5′-GAAGATCTCCGAAGAAGAAGAGGAAGG-3′, and antisense, 5′-GGAATTCTCAGGAGATGACCCTCAG-3′. The amplified DNAs and pEGFPc1 were digested by *Bgl*II-*EcoR*I and ligated together. For the construction of pcDNA3-FLAG-RNH1, specific oligomers and templates were used: sense, 5′-CGGGATCCCATGAGCCTGGACATCCAG-3′ antisense, 5′- GGGGTACCTCAGGAGATGACCCTCAG-3′, and plasmid pSG5L-RNH1. The amplified DNAs and pcDNA3-FLAG [48] were digested by *BamH*I-*Kpn*I and ligated together. The plasmid construct (pcDNA3-pri-miR-21) encoding 212 nt pri-miR-21 was generated by PCR with genomic DNA of HeLa cells as a template and specific oligomers: sense, 5′-CGGGATCCAAATCCTGCCTGACTG TCTGC-3′; and antisense, 5′-GGAATTCTGATTATAAACAATGATGCTGG-3′; The amplified DNAs and pcDNA3 (Invitrogen) were digested by *BamH*I-*EcoR*I and ligated together. For the generation of the Drosha sensors, firefly luciferase gene from pGL3-control (Promega) was inserted between *BamH*I and *Xho*I of pcDNA6.2-GW/EmGFP-miR-neg control (Invitrogen), whose EmGFP was excised by *Dra*I. Then, the pri-miR-21 minigene was inserted into *BamH*I for 5′ sensor or *Xho*I for 3′ sensor. To construct plasmid pEGFPc1-hnRNP A1, the full-length hnRNP A1 cDNA was amplified by PCR from a pGAD424-hnRNP A1 [49] using primers 5′-GGAATTCCATGTCTAAGTCAGAGTCT C-3′ and 5′-CGGGTACCTTAAAATCTTCTGCCACTG-3′. The resulting PCR product was digested with *EcoR*I-*Kpn*I and inserted into the *EcoR*I-*Kpn*I site of pEGFPc1. Plasmids encoding Drosha-WT, Drosha-TN, Lin28-WT, Lin28-TN, pEGFPc1-hnRNP E1, pEGFPc1-YB1, and pEGFPc1-FMRP have been described previously [26], [50], [51], [52]. All constructs were verified by DNA sequencing.

### Real-time PCR of pri-, pre-, and mature miR-21

Quantitative real-time PCR (qRT-PCR) of pri-, pre-, or mature miR-21 was performed as previously described [11]. For the pri- and pre-miR-21 detection, total RNA was extracted from cells by Trizol (Invitrogen). cDNA was synthesized from 1 µg of purified RNA by SuperScript II First-Strand cDNA synthesis system (Invitrogen) according to the manufacturer's instructions. qRT-PCR was performed with QuantiFast SYBR Green PCR master mix (Qiagen) on a Light Cycler 480 (Roche Applied Science). PCR cycling parameters: 94°C for 3 min, and 40 cycles of 94°C for 15 sec, 60°C for 20 sec, 72°C for 40 sec. For detection of mature miRNAs, TaqMan miRNA assay kit (Applied Biosystems) was used according to manufacturer's protocol. For the analysis of pri-miR-21, pre-miR-21, and the normalization control GAPDH, specific oligomers were used as followed. Human pri-miR-21; 5′-TTTTGTTTTGCTTGGGAGGA-3′ and 5′-AGCAGACAGTCAGGCAGGAT-3′. Human pre-miR-21; 5′-TGTCGGGTAGCTTATCAGAC-3′ and 5′-TGTCAGACAGCCCATCGACT-3′. Human GAPDH; 5′-ACCACAGTCCATGCCATCAC-3′ and 5′-TCCACCACCCTGTTGCTGTA-3′. Data analysis was done by using the comparative C_T_ method.

### Antibodies and immunoblots

Anti-PTEN (Cell Signaling Technology, 1/1000), anti-GFP (Santa Cruz Biotech, 1/1000), anti-β-Actin (Santa Cruz Biotech, 1/2000), anti-HA (3F10; Roche Applied Science, 1/1000), anti-RNH1 (Proteintech, 1/1000), anti-Drosha (Abcam, 1/1000), anti-hnRNP A1 (4B10; 1/2000), anti-FLAG (Sigma-Aldrich), anti-DYKDDDDK (Cell Signaling Technology, 1/1000), anti-DDK (4C5; GenDEPOT, 1/1000), and anti-phosphoAKT antibodies (Cell Signaling Technology, 1/1000) were used through the all immunoblot experiments. As a secondary antibody, horseradish peroxidase conjugated anti-rabbit, mouse IgG (Vector Laboratories), and anti-rat IgG (Santa Cruz Biotech) were used.

### *In vitro* pri-miRNA processing assays

*In vitro* pri-miRNA processing assays was performed as previously described [53]. [^32^P]-labeled pri-miR-21 and pri-let-7a-1 were prepared by standard *in vitro* transcription with MEGAshortscript kit (Ambion) in the presence of [α-^32^P]-UTP using human pri-miR-21 and pri-let-7a-1 minigenes as a template. Whole cell extracts were incubated with [^32^P]-labeled pri-miR-21 or pri-let-7a-1 substrates for 90 min at 37°C. Reaction mixtures were subjected to phenol-chloroform extraction and resolved by 8% (w/v) denaturing polyacrylamide gel electrophoresis (PAGE), followed by autoradiography.

### Affinity purification of SFS-tagged protein complexes

Affinity purification of SFS-tagged protein complexes was performed as previously described [47]. To establish cell lines stably expressing SFS-tagged PTEN (SFS-PTEN), 293T cells were transfected with plasmids encoding SFS-PTEN and pGK-puro. 48 hr after transfection, the cells were split at a 1∶10 ratio and cultured in medium containing puromycin (Sigma-Aldrich; 2 µg/ml) for 2 weeks. The individual puromycin-resistant colonies were isolated and screened by immunoblotting with anti-FLAG antibody (Sigma-Aldrich). 10 dishes (100 mm diameter) of confluent 293T cells stably expressing SFS-PTEN were lysed with 3.5 ml TAP lysis buffer [0.5% (v/v) Nonidet P-40, 25 mM Tris-HCl (pH 7.4), 140 mM NaCl, 10 mM NaF, 1 mM Na_3_VO_4_, 1 mM dithiothreitol (DTT), 1 mM phenylmethylsulphonylfluoride (PMSF), 10% (v/v) glycerol, 1 mM β-glycerophosphate, protease inhibitor cocktail (Roche Applied Science) without EDTA, and 1 mM EDTA] on ice for 30 min. After brief lysis, ∼4 ml of lysates further lysed by vortexing for 30 min at 4°C and brief sonication. Crude lysates were cleared by centrifugation at 12,000× g at 4°C for 10 min, and supernatants were incubated with 250 µl streptavidin-conjugated Sepharose beads (GE Healthcare). After 1.5 hr incubation at 4°C, the SFS-PTEN complex was washed two times with 3 ml of TAP lysis buffer. Bead bound protein complexes were eluted with 0.9 ml TAP lysis buffer containing 2 mg/ml biotin (Calbiochem) two times for 30 min at 4°C. The eluents cleared with spin cups cellulose acetate filter (Pierce) and then incubated with 40 µl of S-protein beads (Novagen) for 2 hr at 4°C. The SFS-PTEN complex was washed three times with 0.5 ml of TAP lysis buffer and subjected to SDS-PAGE. Protein bands were visualized by silver staining or coomassie brilliant G (Sigma-Aldrich), excised and digested, and the peptides were analyzed by mass spectrometry.

### LC-MS/MS and database search

SDS-PAGE gels containing proteins of interest were excised, destained with 50% acetonitrile in 0.1 M ammonium bicarbonate, and dried in a SpeedVac evaporator. Dried gel pieces were re-swollen with 30 µl of 25 mM sodium bicarbonate, pH 8.8, containing 50 ng trypsin (Promega) at 37°C overnight. Samples were desalted using Zip-Tips C18 (Millipore), and dissolved in 10 µl 2% acetonitrile in 0.1% formic acid. Analysis was performed using a LTQ XL linear ion trap mass spectrometer system (Thermo Fisher Scientific) in Proteomics Core, National Cancer Center, Korea. The mass spectrometry was set for NSI in positive mode. A syringe pump was used to introduce the calibration solution for automatic tuning and calibration of the LTQ in NSI positive ion mode. Infusion of digested samples (trypsin) into the ionization source of the mass spectrometry was accomplished with liquid chromatographic separation. The spray voltage was set at +1.1 kV, while the temperature of the capillary was set at 200°C, the capillary voltage was set at +20 V and the tube lens voltage was set at +100 V. The auxiliary gas was set to zero. Full scan experiments were performed to linear trap in the range m/z 150–2000. Systematic MS/MS experiments were performed by changing the relative collision energy and monitoring the intensities of the fragment ions. All MS/MS samples were analyzed using Sequest (Thermo Fisher Scientific; version v.27, rev. 11). Sequest was set up to search the uniprot_sprot database and IPI human database assuming the digestion enzyme trypsin. Sequest was searched with a fragment ion mass tolerance of 1.00 Da and a parent ion tolerance of 1.2 Da. Oxidation of methionine was specified in Sequest as a variable modification.

### Immunoprecipitation, mRNP-immunoprecipitation, and semi-quantitative RT-PCR

Immunoprecipitation (IP) was performed as previously described [48]. Cells were crushed in IP buffer [150 mM NaCl, 25 mM HEPES-KOH (pH 7.5), 10% (v/v) glycerol, 1 mM MgCl_2_, 2 mM sodium orthovanadate, 2 mM β-glycerophosphate, 1 mM PMSF, 1 mM DTT, 2 mM EDTA, 0.5% Triton X-100, and 1× protease inhibitor cocktail (Roche Applied Science)]. After brief sonication and incubation on ice, lysates were centrifuged at 12,000× g for 5 min to remove insoluble materials. The lysates were then incubated with anti-FLAG M2 affinity agarose gels (Sigma-Aldrich) for 2 hr at 4°C. The collected beads were then washed four times with washing buffer (0.05% Triton X-100 IP buffer without protease inhibitor cocktail) and boiled in SDS sample buffer for immunoblot analysis. mRNP-IP was performed by the same strategy as protein IP except including RNaseOUT (Invitrogen) in the reaction mixture. The lysates were then incubated with anti-FLAG M2 affinity agarose gels for 1 hr at 4°C. The collected beads were then washed four times with washing buffer and the total RNA was isolated using TRI-Reagent (Molecular Research Center, Inc.) according to the manufacturer's instructions. cDNAs were synthesized using ImProm-II reverse transcription system (Promega) with oligo(dT)_20_ (Bioneer Co.) according to the manufacturer's instructions. Reaction parameters are as followed; 30 cycles of 94°C for 30 sec, 55°C for 30 sec, and 72°C for 30 sec. The PCRs were performed with specific oligomers (Bioneer Co.) for pri-miR-21 and pri-miR-29a with e-Taq (Solgent Co.): pri-miR-21, 5′-CGGGATCCAAATCCTGCCTGACTGTCTGC-3′ and 5′-GGAATTCTGATTA TAAACAATGATGCTGG-3′; pri-miR-29a, 5′-CGGGATCCAAGAGCCCAATGTATGCTGG-3′ and 5′-GG AATTCAACGGTCACCAATACATTTCC-3′. The PCR products were analyzed on the 1% agarose gel.

### Transfection and dual luciferase assays

The Drosha sensors or control plasmid were co-transfected with pRL-TK (Promega) into 293T cells by using Lipofectamine 2000 (Invitrogen) according to the manufacturer's instructions. After 48 h, cells were lysed with passive lysis buffer (Promega). Aliquots of lysates were analyzed by dual luciferase reporter assay system (Promega). All the signals from firefly luciferase of sensors were first normalized with that from *Renilla* luciferase. Then the normalized values were re-normalized with the normalized value of signals from control plasmid.

### Small RNA Northern blot analysis

Total RNA isolation and Northern blotting was carried out following standard procedures [53]. 50 µg of total RNA was separated on 12% SequaGel (National Diagnostics) and transferred to Biodyne nylon transfer membranes (Pall Co.). The membrane was hybridized overnight at 37°C with a [^32^P] 5′-end-labeled oligonucleotide probe (anti-miR-21; 5′-TCAACATCAGTCTGATAAGCTA-3′) in ExpressHyb solution (Clontech) and then washed according to standard procedures. Radioactive signals were scanned by the BAS-2500 analyzer (Fujifilm).

### siRNAs

To knockdown the following human proteins, siRNAs were synthesized by Bioneer Co.; human Drosha 5′-CAGCAAUGGAUGCGCUUGA-3′ and human RNH1 5′-CUGCAUAUCCUAGGUUUGA -3′. As a negative control, siRNA specific for GFP was used; 5′-GUUCAGCGUGUCCGGCGAG-3′.

### Confocal fluorescence microscopy

293 cells were grown on cover slips which were coated with 0.5 mg/ml poly-L-Lysine (Sigma-Aldrich). Plasmids encoding GFP, GFP-WT-RNH1, and GFP-NLS-RNH1 were transfected to the 293 cells by using Lipofectamine 2000 according to the manufacturer's instructions. After 48 h, 293 cells were fixed in 3.75% paraformaldehyde (Sigma-Aldrich) and the cover slips were washed twice with Dulbecco's phosphate-buffered saline were mounted with Vectashield mounting medium (Vector Laboratories). Fluorescent images were acquired with confocal laser scanning microscopy system (model LSM510 meta; Carl Zeiss) and Axio Observer Z1 (Carl Zeiss) using GFP and DAPI filters. The confocal system software and Axiovision software were used to capture and store the images. Confocal images for GFP, GFP-WT-RNH1, and GFP-NLS-RNH1 were shown in green, while the nuclei were shown by blue. Merged images of green and blue are shown in sky-blue.

### Statistical analysis of data

All values were reported as mean±standard error of means (s.e.m.). Differences were assessed by the two-tailed Student's t-test using Excel software (Microsoft). *P*<0.05 was considered as statistically significant.

## References

[1] Bartel DP (2009). MicroRNAs: target recognition and regulatory functions.. Cell.

[2] Filipowicz W, Bhattacharyya SN, Sonenberg N (2008). Mechanisms of post-transcriptional regulation by microRNAs: are the answers in sight?. Nat Rev Genet.

[3] Garofalo M, Croce CM (2011). microRNAs: Master regulators as potential therapeutics in cancer.. Annu Rev Pharmacol Toxicol.

[4] Farazi TA, Spitzer JI, Morozov P, Tuschl T (2011). miRNAs in human cancer.. J Pathol.

[5] Kumar MS, Lu J, Mercer KL, Golub TR, Jacks T (2007). Impaired microRNA processing enhances cellular transformation and tumorigenesis.. Nat Genet.

[6] Thomson JM, Newman M, Parker JS, Morin-Kensicki EM, Wright T (2006). Extensive post-transcriptional regulation of microRNAs and its implications for cancer.. Genes Dev.

[7] Kim VN, Han J, Siomi MC (2009). Biogenesis of small RNAs in animals.. Nat Rev Mol Cell Biol.

[8] Suzuki HI, Miyazono K (2011). Emerging complexity of microRNA generation cascades.. J Biochem.

[9] Siomi H, Siomi MC (2010). Posttranscriptional regulation of microRNA biogenesis in animals.. Mol Cell.

[10] Krol J, Loedige I, Filipowicz W (2010). The widespread regulation of microRNA biogenesis, function and decay.. Nat Rev Genet.

[11] Davis BN, Hilyard AC, Lagna G, Hata A (2008). SMAD proteins control DROSHA-mediated microRNA maturation.. Nature.

[12] Yamagata K, Fujiyama S, Ito S, Ueda T, Murata T (2009). Maturation of microRNA is hormonally regulated by a nuclear receptor.. Mol Cell.

[13] Trabucchi M, Briata P, Garcia-Mayoral M, Haase AD, Filipowicz W (2009). The RNA-binding protein KSRP promotes the biogenesis of a subset of microRNAs.. Nature.

[14] Suzuki HI, Yamagata K, Sugimoto K, Iwamoto T, Kato S (2009). Modulation of microRNA processing by p53.. Nature.

[15] Selcuklu SD, Donoghue MT, Spillane C (2009). miR-21 as a key regulator of oncogenic processes.. Biochem Soc Trans.

[16] Krichevsky AM, Gabriely G (2009). miR-21: a small multi-faceted RNA.. J Cell Mol Med.

[17] Kwak HJ, Kim YJ, Chun KR, Woo YM, Park SJ (2011). Downregulation of Spry2 by miR-21 triggers malignancy in human gliomas.. Oncogene.

[18] Pflieger D, Bigeard J, Hirt H (2011). Isolation and characterization of plant protein complexes by mass spectrometry.. Proteomics.

[19] Volkel P, Le Faou P, Angrand PO (2010). Interaction proteomics: characterization of protein complexes using tandem affinity purification-mass spectrometry.. Biochem Soc Trans.

[20] Kim S, Hwang do W, Lee DS (2009). A study of microRNAs in silico and in vivo: bioimaging of microRNA biogenesis and regulation.. FEBS J.

[21] Guil S, Caceres JF (2007). The multifunctional RNA-binding protein hnRNP A1 is required for processing of miR-18a.. Nat Struct Mol Biol.

[22] Michlewski G, Caceres JF (2010). Antagonistic role of hnRNP A1 and KSRP in the regulation of let-7a biogenesis.. Nat Struct Mol Biol.

[23] Viswanathan SR, Daley GQ, Gregory RI (2008). Selective blockade of microRNA processing by Lin28.. Science.

[24] Jeong SH, Wu HG, Park WY (2009). LIN28B confers radio-resistance through the posttranscriptional control of KRAS.. Exp Mol Med.

[25] Yang X, Lin X, Zhong X, Kaur S, Li N (2010). Double-negative feedback loop between reprogramming factor LIN28 and microRNA let-7 regulates aldehyde dehydrogenase 1-positive cancer stem cells.. Cancer Res.

[26] Heo I, Joo C, Cho J, Ha M, Han J (2008). Lin28 mediates the terminal uridylation of let-7 precursor MicroRNA.. Mol Cell.

[27] Furia A, Moscato M, Cali G, Pizzo E, Confalone E (2011). The ribonuclease/angiogenin inhibitor is also present in mitochondria and nuclei.. FEBS Lett.

[28] Hollander MC, Blumenthal GM, Dennis PA (2011). PTEN loss in the continuum of common cancers, rare syndromes and mouse models.. Nat Rev Cancer.

[29] Chalhoub N, Baker SJ (2009). PTEN and the PI3-kinase pathway in cancer.. Annu Rev Pathol.

[30] Meng F, Henson R, Wehbe-Janek H, Ghoshal K, Jacob ST (2007). MicroRNA-21 regulates expression of the PTEN tumor suppressor gene in human hepatocellular cancer.. Gastroenterology.

[31] Vinciguerra M, Sgroi A, Veyrat-Durebex C, Rubbia-Brandt L, Buhler LH (2009). Unsaturated fatty acids inhibit the expression of tumor suppressor phosphatase and tensin homolog (PTEN) via microRNA-21 up-regulation in hepatocytes.. Hepatology.

[32] Inui M, Martello G, Piccolo S (2010). MicroRNA control of signal transduction.. Nat Rev Mol Cell Biol.

[33] Song MS, Carracedo A, Salmena L, Song SJ, Egia A (2011). Nuclear PTEN regulates the APC-CDH1 tumor-suppressive complex in a phosphatase-independent manner.. Cell.

[34] Song MS, Salmena L, Carracedo A, Egia A, Lo-Coco F (2008). The deubiquitinylation and localization of PTEN are regulated by a HAUSP-PML network.. Nature.

[35] Trotman LC, Wang X, Alimonti A, Chen Z, Teruya-Feldstein J (2007). Ubiquitination regulates PTEN nuclear import and tumor suppression.. Cell.

[36] Li AG, Piluso LG, Cai X, Wei G, Sellers WR (2006). Mechanistic insights into maintenance of high p53 acetylation by PTEN.. Mol Cell.

[37] Shen WH, Balajee AS, Wang J, Wu H, Eng C (2007). Essential role for nuclear PTEN in maintaining chromosomal integrity.. Cell.

[38] Planchon SM, Waite KA, Eng C (2008). The nuclear affairs of PTEN.. J Cell Sci.

[39] Bella J, Hindle KL, McEwan PA, Lovell SC (2008). The leucine-rich repeat structure.. Cell Mol Life Sci.

[40] Teplova M, Wohlbold L, Khin NW, Izaurralde E, Patel DJ (2011). Structure-function studies of nucleocytoplasmic transport of retroviral genomic RNA by mRNA export factor TAP.. Nat Struct Mol Biol.

[41] Dickson KA, Haigis MC, Raines RT (2005). Ribonuclease inhibitor: structure and function.. Prog Nucleic Acid Res Mol Biol.

[42] Fukuda T, Yamagata K, Fujiyama S, Matsumoto T, Koshida I (2007). DEAD-box RNA helicase subunits of the Drosha complex are required for processing of rRNA and a subset of microRNAs.. Nat Cell Biol.

[43] Gruber JJ, Zatechka DS, Sabin LR, Yong J, Lum JJ (2009). Ars2 links the nuclear cap-binding complex to RNA interference and cell proliferation.. Cell.

[44] Sabin LR, Zhou R, Gruber JJ, Lukinova N, Bambina S (2009). Ars2 regulates both miRNA- and siRNA- dependent silencing and suppresses RNA virus infection in Drosophila.. Cell.

[45] Wu H, Sun S, Tu K, Gao Y, Xie B (2010). A splicing-independent function of SF2/ASF in microRNA processing.. Mol Cell.

[46] Rau F, Freyermuth F, Fugier C, Villemin JP, Fischer MC (2011). Misregulation of miR-1 processing is associated with heart defects in myotonic dystrophy.. Nat Struct Mol Biol.

[47] Kim H, Huang J, Chen J (2007). CCDC98 is a BRCA1-BRCT domain-binding protein involved in the DNA damage response.. Nat Struct Mol Biol.

[48] Kim JH, Richter JD (2006). Opposing polymerase-deadenylase activities regulate cytoplasmic polyadenylation.. Mol Cell.

[49] Kim JH, Hahm B, Kim YK, Choi M, Jang SK (2000). Protein-protein interaction among hnRNPs shuttling between nucleus and cytoplasm.. J Mol Biol.

[50] Lee Y, Ahn C, Han J, Choi H, Kim J (2003). The nuclear RNase III Drosha initiates microRNA processing.. Nature.

[51] Cho S, Park SM, Kim TD, Kim JH, Kim KT (2007). BiP internal ribosomal entry site activity is controlled by heat-induced interaction of NSAP1.. Mol Cell Biol.

[52] Cho S, Kim JH, Back SH, Jang SK (2005). Polypyrimidine tract-binding protein enhances the internal ribosomal entry site-dependent translation of p27Kip1 mRNA and modulates transition from G1 to S phase.. Mol Cell Biol.

[53] Lee Y, Kim VN (2007). In vitro and in vivo assays for the activity of Drosha complex.. Methods Enzymol.

